# Reference Intervals for Hematological Inflammatory Ratios in Healthy Dogs

**DOI:** 10.3390/ani15233376

**Published:** 2025-11-21

**Authors:** Paula F. Navarro, Miquel Monroig, Laura Gil-Vicente

**Affiliations:** 1 Facultad de Veterinaria y Ciencias Experimentales, Universidad Católica de Valencia San Vicente Mártir, 46001 Valencia, Spain; 2 Escuela de Doctorado, Universidad Católica de Valencia San Vicente Mártir, 46001 Valencia, Spain; 3 Hospital Veterinario UCV, Universidad Católica de Valencia San Vicente Mártir, 46018 Valencia, Spain

**Keywords:** canine, hematological inflammatory ratios, MLR, NLR, PLR, reference interval, SII

## Abstract

Hematological inflammatory ratios such as the neutrophil-to-lymphocyte (NLR), monocyte-to-lymphocyte (MLR), platelet-to-lymphocyte (PLR), and systemic inflammatory index (SII) have recently demonstrated prognostic value in acute inflammatory diseases, neoplastic conditions, hepatic and gastrointestinal pathologies, as well as acting as biomarkers for the early detection of tumors and cardiorespiratory diseases. In addition, they can be easily calculated and have no additional cost. The purpose of this study was to establish reference ranges for these ratios in the canine population, thereby providing support for clinical practice. Data was collected from previous studies. The dogs included in the study had to have anamnesis and physical examination without abnormalities and within reference limits in laboratory results. A total of 156 samples were included and divided by sex and age. The following reference intervals were established: 1.3–7.1 for NLR; 0.1–0.3 for MLR; 40.4–271.1 for PLR, and 224.6–2191.7 for SII. Statistical significant difference was found between sexes and NLR, with males showing a higher ratio than females. Regarding age, statistical significance was found between juveniles and senior dogs for NLR. PLR and SII showed higher ratios in senior dogs compared to adults and juveniles. This study may serve as a guide in clinical practice for the interpretation of hematological inflammatory ratios.

## Simple Summary

In the present study, reference intervals for hematological ratios in dogs were established. Hematological inflammatory ratios, such as the neutrophil-to-lymphocyte (NLR), monocyte-to-lymphocyte (MLR), platelet-to-lymphocyte (PLR), and systemic inflammatory index (SII), are widely used because they are easily calculated from a complete blood count and involve no additional cost. Hematological ratios have recently demonstrated prognostic value in acute inflammatory diseases, neoplastic conditions, hepatic and gastrointestinal pathologies, as well as acting as biomarkers for the early detection of tumors and cardiorespiratory diseases. The following reference intervals were established: 1.3–7.1 for NLR; 0.1–0.3 for MLR; 40.4–271.1 for PLR, and 224.6–2191.7 for SII. This may represent a useful tool in clinical practice, enabling rapid comparison and supporting clinicians in prognostic assessment of certain diseases, as well as potentially serving as a predictive biomarker.

## 1. Introduction

The use of novel biomarkers as an available tool in small animal practice has increased in recent years in veterinary medicine [1]. Depending on their use, biomarkers can be categorized as risk, diagnostic, monitoring, predictive, prognostic, response, or safety biomarkers [2].

Hematological biomarker ratios such as neutrophil-to-lymphocyte (NLR), monocyte-to-lymphocyte (MLR) and platelet-to-lymphocyte (PLR) can be easily obtained by performing a complete blood count (CBC) and have been used to determine prognosis in acute inflammatory diseases such as peritonitis, pancreatitis or acute diarrhea [3,4,5,6,7,8], neoplastic conditions [9,10,11,12], and cardiovascular, intestinal or hepatic pathologies [13,14,15,16,17,18]. These hematological ratios have proven to be more effective than individual hematological parameters in assessing some systemic responses [4,5,6].

In one study, dogs with systemic inflammatory response syndrome and septicemia showed a lower NLR compared to the non-septic dogs [4]. In dogs with chronic enteropathies, NLR has been useful to subclassify the origin of the enteropathy and has also been useful as an inflammatory marker [17,18]. Furthermore, it can also be used as a predictive marker, as in another study, NLR was higher in dogs affected by a portosystemic shunt that required more than one intervention [14]. NLR also has a prognostic value in mammary tumors, where higher ratios are related to a lower survival rate [12]. In dogs affected by soft tissue sarcoma, NLR appears to be higher than in dogs with benign soft tissue tumors [19]. NLR has also been found significantly higher in dogs with oropharyngeal tumors compared to dogs with periodontitis or healthy dogs [20]. In one study comparing healthy and sick seropositive dogs infected by *Leishmania infantum*, NLR was higher in moderate to severe stages of the disease compared to the healthy seropositive and seronegative dogs. Additionally, in this study, MLR values were higher in seropositive dogs with hypoalbuminemia [21]. In dogs with diffuse large B-cell lymphoma, lower MLR was found to be a useful predictor of shorter time-to-progression and survival [11]. In another study conducted by Marchesi et al. on dogs with inflammatory bowel disease (IBD), an MRL above the reference range is highly indicative of the disease [22]. In dogs with primary immune-mediated hemolytic anemia, a low MLR in combination with a high NLR was associated with a poorer prognosis [23].

In a study involving puppies affected by parvovirosis, PLR was higher in non-survivors [5]. Similarly, in dogs with acute pancreatitis, this ratio was higher than in the control group and linked to a longer recovery [6]. In dogs with chronic enteropathy, PLR could be considered a potential marker of treatment efficacy [24]. Also, this marker decreases during treatment time [13]. In addition, PLR could be used as a screening tool for malignancy in nasal tumors [25].

The systemic inflammation index (SII), which can be calculated by multiplying the total neutrophil count by the total platelet count and dividing by the total lymphocyte count, has been widely used in human medicine mostly as a biomarker predictor for certain tumors as well as respiratory and cardiovascular disease [26,27,28,29]. In veterinary medicine, few studies have evaluated its potential use as a novel inflammation marker of prognosis in inflammatory or neoplastic diseases [13,30]. In one study, dogs that survived leptospirosis infection showed higher SII values than healthy dogs or dogs that died from the disease [31]. In dogs with leishmaniosis, SII tended to increase as the disease worsened [32]. Dogs with a high body condition score also had higher SII values [33].

To the author’s knowledge, there is no information regarding hematological ratios reference intervals in a large population of dogs, although its use has been proven to be more effective than the use of individual indicators. The aims of this study were to establish reference intervals for the NLR, MLR, PLR, and SII in a large population of dogs, and to determine if there were significant differences between age groups or sexes.

## 2. Materials and Methods

This was a retrospective observational study, using samples collected from twenty-three veterinary centers, including first opinion veterinary clinics and reference veterinary hospitals, between 2018 and 2024. The study was conducted in the Mediterranean area of Spain, including outdoor and indoor dogs; data such as age or sex were included randomly. Preventive care information, such as deworming or vaccination, was not available for all patients and, therefore, was not included in the study. To be included in the study, dogs had to have anamnesis and physical examination without abnormalities and obtain laboratory results within reference limits, including negative *Leishmania infantum* antibodies titers. Physiological or subclinical age-related changes, such as periodontitis conditions or joint degeneration, were not considered diseases in dogs over 8 years of age.

Data were collected from previous studies by the same investigation group, and the examined sample was divided into two groups by age range (juvenile from 0 to 1 years old; adult from 1 to 8 years old; senior from 8 years onwards) and by sex (males and females).

All samples were processed by the same reference laboratory. The analysis included a complete blood count (CBC; Celltac Alpha VET MEK-6550; Nihon Khoden, Rosbach, Germany) with blood smear evaluation (Figure S1). Biochemical analytes measured included creatinine, urea, alanine aminotransferase, and total serum proteins (CS 300 analyzer; Dirui, Jilin, China). Serum electrophoretograms were run by CE (Minicap instrument; Sebia, Lisses, France). Serum of all animals was also tested for antibodies to *Leishmania infantum* by an immunofluorescent antibody test (IFAT) (Axio Scope HBO 50 microscope; Zeiss, Madrid, Spain) because this infectious agent is highly prevalent in the study area.

Hematological ratios were calculated by dividing the total neutrophil count, the total monocyte count, and the total platelet count by the total lymphocyte count. The units reported by the reference laboratory for all white blood cells (WBCs) and platelets were the same (K/µL). Dogs with platelet aggregates were detected by the blood smear and excluded from the PLR and SII. SII was calculated by multiplying the total neutrophil count by the total platelet count and dividing by the total lymphocyte count.

Outliers of each hematological value were identified using the Reference Value Advisor macro (v. 2.0; Redmon, WA, USA). Histograms of each hematological ratio were assessed to identify potential outliers. Tukey interquartile fences and the Dixon outlier range statistic were used to identify true and suspected outliers. True outliers were deleted, whereas suspected outliers were retained following the American Society for Veterinary Clinical Pathology (ASVCP) guidelines [34]. RIs were obtained (Reference value advisor v.2.0; Microsoft) through the nonparametric method, given that the population size was large enough (>120). Information regarding data distribution was not necessary because nonparametric methods were used. Moreover, 95% RIs were calculated with 90% confidence intervals (CIs) for reference limits according to the ASVCP 2012 guidelines.

Statistical analysis for each subgroup was performed using R version 4.4.1 (R Development Core Team, Vienna, Austria). The Shapiro–Wilk test was used for each group to test the hypothesis of normality. Outliers were detected by box-plot and eliminated if considered aberrant observations. Since the data did not follow a normal distribution, the Mann–Whitney U test was used to establish differences between the two groups. The Kruskal–Wallis test was used to compare more than two independent groups. A post hoc analysis of statistically significant associations was conducted using Dunn’s test with Bonferroni correction. Results were considered statistically significant if *p* < 0.05. The plots were created using Matplotlib version 3.8.0 software (Matplotlib Development Team, Austin, TX, USA).

## 3. Results

**Figure 1:**
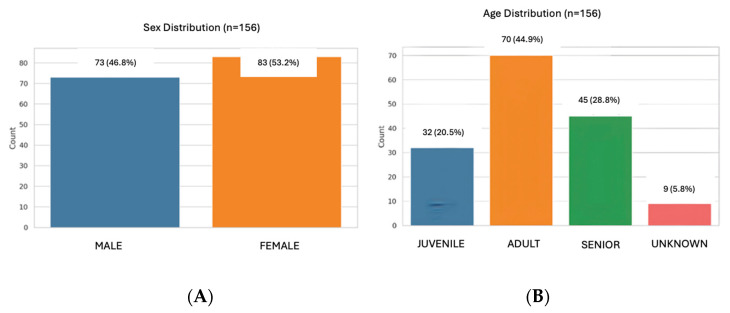
Distribution of the population by sex (**A**) and age (**B**).

**Figure 2:**
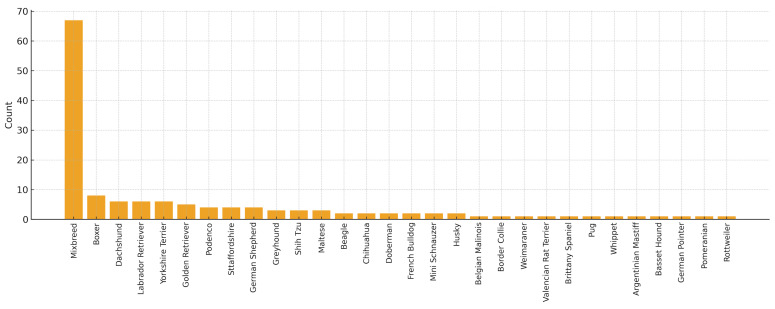
Description of the population according to breed included in the study.

The study population included 156 samples from healthy dogs, 73 males (46.8%) and 83 females (53.2%). A total of 32 dogs aged 0 months to 1 year (20.5%), 70 dogs aged 1 to 8 years (44.9%), and 45 dogs older than 8 years were included (28.8%) (Figure 1). Several breeds were included: Boxer (*n* = 8), Labrador Retriever (*n* = 6), Dachshund (*n* = 6), Yorkshire Terrier (*n* = 6), Golden Retriever (*n* = 5), Podenco (*n* = 4), Sttaffordshire Bull Terrier (*n* = 4), German Sheperd (*n* = 4), Greyhound (*n* = 3), Shih Tzu (*n* = 3), Maltese (*n* = 3), Beagle (*n* = 2), French Bulldog (*n* = 2), Chihuahua (*n* = 2), Doberman (*n* = 2), Mini Schnauzer (*n* = 2), Siberian Husky (*n* = 2), Belgian Malinois (*n* = 1), Boder Collie (*n* = 1), Valencian Terrier (*n* = 1), Brittany Spaniel (*n* = 1), Pug (*n* = 1), Whippet (*n* = 1), Weimaraner (*n* = 1), Argentinian Mastiff (*n* = 1), Basset Hound (*n* = 1), Pointer (*n* = 1), Pomeranian (*n* = 1), Rottweiler (*n* = 1). A total of 67 dogs were mix breed. In 13 dogs, the breed was not registered (Figure 2).

PLR and SII were calculated based on a 124 population, as platelet aggregates were found on 32 blood smears and excluded from these parameters.

From the PLR and SII, 1 outlier was found to be aberrant and deleted.

RIs were obtained for the different ratios as follows: 1.3–7.1 for NLR; 0.1–0.3 for MLR; 40.4–271.1 for PLR, and 224.6–2191.7 for SII (Table 1).

**Figure 3:**
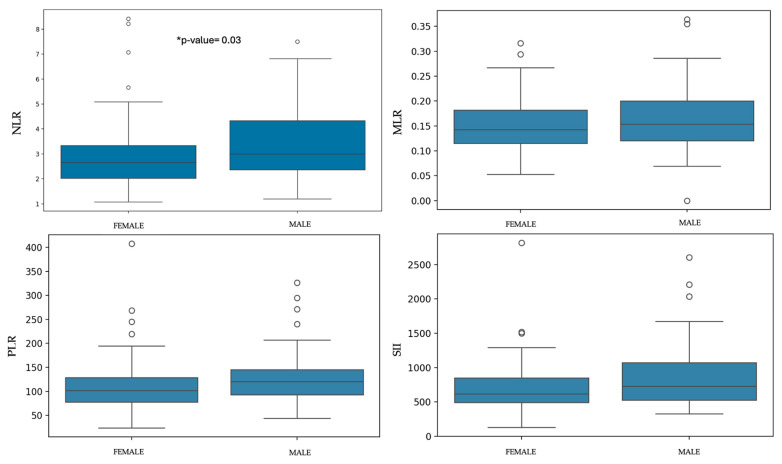
Box-plots of each ratio comparing females and males. * Statistically significant differences were observed with higher values in males than in females.

**Figure 4:**
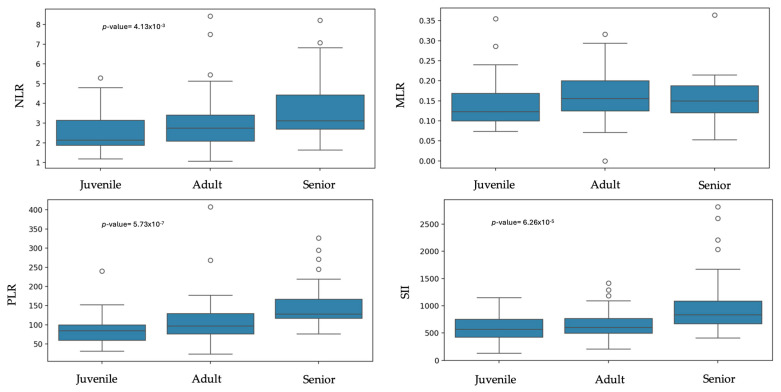
Box plots of the inflammatory ratios across age groups.

Statistically significant difference were found between sexes and NLR, with males showing a higher ratio than females (*p*-value = 0.03). No significant differences were observed between males and females for MLR, PLR, and SII (Table 2 and Figure 3). Regarding age, statistically significant differences were found between juveniles and senior dogs for NLR (*p*-value = 4.13 × 10^−3^). PLR and SII values were significantly higher in senior dogs compared to adult and juvenile dogs (PLR: *p*-value = 5.73 × 10^−7^; SII: *p*-value = 6.26 × 10^−5^). MLR showed no statistical differences between groups (Table 3 and Figure 4).

## 4. Discussion

This study establishes hematological ratios with a high confidence level in a large population of healthy dogs.

Generally, most studies conducted to date have compared a population of sick dogs with relatively small samples of healthy controls. The largest sample size study compares the NLR in dogs with inflammatory bowel disease with 150 healthy dogs, in which the NLR in healthy dogs showed a range between 1.1 and 13.3 [18]. The upper reference limit in our study is lower (7.1); this difference could be due to the age or breed of the dogs included in the study, since previous studies have confirmed variations in these parameters, such as a lower lymphocyte count or a high neutrophil count in older dogs [35]. The type of analyzer used in this study is not specified, which could potentially contribute to differences between studies. In another study, RI for NLR in a group of 44 healthy dogs ranged from 1.0 to 4.1, but it was also correlated with age, with no further specifications of the dogs included. In the present study, 45 dogs were older than 8 years, which could be associated with the observed increase in the upper limit of the reference range [17]. In addition, an age-related increase in neutrophils is commonly observed [36,37]. NLR has been found significantly higher in dogs with myxomatous mitral valve disease, mammary tumors, leishmaniosis, and oral tumors [12,20,21,38].

The RI for MLR in this study was 0.1 to 0.3, consistent with other studies using a small number of dogs as a control population. These studies also showed statistically significant differences between healthy dogs and dogs with severe myxomatous valve disease, leishmaniosis, or inflammatory bowel disease [21,22,38,39]. These results may be attributable to the monocytosis and lymphopenia secondary to inflammation, although it should be taken into account that stress situations could cause these alterations [18,40].

The RI for PLR was 40.4 to 271.1, this result is consistent with the control groups of other studies [21,31,39], however they differ from the findings of a study in dogs where values higher than 131.6 were used as a predictor of IBD, although in this study blood smears were not examined, raising the possibility that the platelet count was actually higher due to the presence platelet aggregates and therefore the PLR would also be higher [22]. Another study conducted in dogs reported that PLR higher than 285 could be consistent with hypercortisolism; these results are in agreement with our results, as the PLR cutoff associated with hypercortisolism (>285) is higher than the upper reference limit established in our population [41].

SII is significantly higher in studies conducted in dogs with obesity, leishmaniasis, enteropathies, diabetes mellitus, and vector-borne diseases [13,31,33,42,43]. In the present study, the RI was 224.6–2191.7. This reference range is wider than that reported in another study (52.93–1503) with a control population of 20 dogs, which may be attributable to the smaller control population or to other factors such as body condition, since it has been shown to increase this index [33], and was not a variable considered in the present study; or the age of the dogs included in the study as it is a factor that could lead to changes in the CBC [35,37].

Significant differences were found between males and females in this study for the NLR. This finding is consistent with previous studies in both humans and dogs, which reported higher absolute neutrophil counts in males compared to females [35,44,45]. This difference may be explained by the influence of sex hormones in intact males, as androgens might promote the myeloid lineage cells [35]; however, reproductive status was not considered in the present study.

Age was identified as a statistically significant factor for NLR, with lower values in dogs from 0 to 1 year old than in seniors, and for PLR and SII, which were significantly higher in seniors compared with adults and juveniles. This could be because neutrophils increase in adulthood, probably as a result of physiological or subclinical age-related changes, such as periodontal disease or joint degeneration, and by the decline in lymphocytes resulting from a reduced immune response [35,36,37]. Platelet count also has a positive correlation with aging [35,37,46], probably due to inflammation or thrombocytosis secondary to an epinephrine response [35,36]. These data will be relevant for the individualization of ratios in diseased patients, taking into account both sex and age.

Limitations of this study are that certain variables, potentially having a significant impact on certain results, such as reproductive status and body condition, were not considered. In addition, age-related conditions such as periodontitis and joint degeneration were not considered as a disease in the group of dogs over 8 years of age, which could have influenced some of the hematological parameters. Another limitation of the study, given that the patients were obtained from previous studies, is the lack of standardization in the information collected and physical examination, data that could potentially have affected the results. Furthermore, to rule out any subclinical disease, other tests such as urinalysis, coprological, or other serological tests could have been performed. However, despite the increasing study of these hematological ratios in veterinary medicine as both diagnostic and prognostic markers, this is the first study to present reference ranges in a large population of dogs, including differences between males and females and across different age groups, which may be of great clinical relevance for certain pathologies. Future lines of investigation could include prospective studies with a better-defined population and standardized clinical evaluations, including parameters such as body condition, vaccination and deworming status, reproductive status, and age-related conditions.

## 5. Conclusions

Reference intervals for NLR, MLR, PLR, and SII can be used as a guide in clinical practice to help in the diagnosis, monitoring, and prognosis of inflammatory or neoplastic diseases in canine patients, providing clinicians with accessible, cost-free, and clinically relevant biomarkers derived from a routine complete blood count. This study also reveals the importance of taking individual factors such as age or gender into account when evaluating these parameters to avoid misinterpretation and to improve diagnostic accuracy. These reference intervals represent a meaningful step forward for integrating hematological inflammatory ratios into everyday clinical decision-making, supporting early detection, disease monitoring, and prognostic assessment across a wide range of conditions. Future research should build on this foundation by evaluating these ratios in specific pathological contexts and disease severities, with the goal of refining their use as reliable, evidence-based biomarkers in veterinary medicine.

## Figures and Tables

**Table 1:** Reference intervals for hematological inflammatory ratios in healthy dogs.

Analyte	*n*	Median	RI	IQR	LRL (90%)	URL (90%)	Method
NLR	156	2.86	1.3–7.1	1.48	1.1–1.6	5.7–8.4	NP
MLR	156	0.14	0.1–0.3	0.07	0.0–0.1	0.3–0.4	NP
PLR	123	110.55	40.4–271.1	50.98	24.1–51.0	219.4–326.0	NP
SII	123	658	224.6–2191.7	458.49	131.3–380.0	1518.7–2817.5	NP

IQR = interquartile range; LRL = lower reference limit; NP = nonparametric; RI = reference interval; URL = upper reference limit.

**Table 2:** Comparison of the different inflammatory ratios between females and males.

Analyte	*n*	Female Median (25th and 75th Percentiles)	Male Median (25th and 75th Percentiles)	*p*-Value
NLR	83 (F) 73 (M)	2.65 (2.01–3.34)	3.0 (2.35–4.33) *	0.03
MLR	83 (F) 73 (M)	0.143 (0.11–0.18)	0.154 (0.12–0.20)	0.12
PLR	64 (F) 59 (M)	102.17 (77.93–128.95)	120.24 (92.90–145.62)	0.11
SII	64 (F) 59 (M)	617.25 (490.16–851.95)	731.67 (526.47–1074.49)	0.07

F = female; M = male. * Statistically significant differences were observed with higher values in males than in females.

**Table 3:** Age-related comparison of hematological inflammatory ratios.

Analyte	*n*	Juveniles’ Median (25th and 75th Percentiles)	Adults’ Median (25th and 75th Percentiles)	Seniors’ Median (25th and 75th Percentiles)	*p*-Value
NLR	32 (J) 70 (A) 45 (S)	2.15 (1.87–3.14)	2.75 (2.09–3.42)	* 3.12 (2.69–4.43)	4.13 × 10^−3^
MLR	32 (J) 70 (A) 45 (S)	0.12 (0.1–0.16)	0.15 (0.12–0.19)	0.15 (0.12–0.18)	0.08
PLR	23 (J) 54 (A) 38 (S)	84.72 (60.11–100.16)	97.04 (76.67–129.61)	** 128.06 (117.03–166.74)	5.73 × 10^−7^
SII	23 (J) 54 (A) 38 (S)	570.14 (426.78–753.73)	604.75 (497.5–768.24)	** 834.85 (669.67–1089)	6.26 × 10^−5^

J = Juvenile; A = Adult; S = Senior. * Statistically significant differences were observed with lower values in juveniles than in adults and seniors; ** statistically significant differences were observed, with higher values in seniors than in juveniles and adults.

## Data Availability

Data are contained within the article.

## Supplementary Materials

The following supporting information can be downloaded at: https://www.mdpi.com/article/10.3390/ani15233376/s1, Figure S1. Complete analysis with reference values.

## Abbreviations

The following abbreviations are used in this manuscript:

NRL	Neutrophil-to-lymphocyte ratio
MRL	Monocyte-to-lymphocyte ratio
PLR	Platelet-to-lymphocyte ratio
SII	Systemic inflammatory index
