# Supplementary material for: Reference Intervals for Hematological Inflammatory Ratios in Healthy Dogs
Source: Animals (Basel). 2025 Nov 21;15(23):3376. doi: 10.3390/ani15233376 (PMC12691325; doi:10.3390/ani15233376)
Supplement: Supplementary file 1 [file animals-15-03376-s001.zip › animals-3953273-supplementary.pdf]

Datos personales

Centro veterinario

Dr. / Dra.

Nº Muestra	Fecha recepción	Fecha impresión	Especie
	**30/08/2024**	**02/09/2024**	**Perro**

PRUEBA	RESULTADO		VAL. DE REFERENCIA
**HEMOGRAMA**			
·LEUCOCITOS	8,0	k/uL.	5,5 - 17 K/uL.
**Serie Roja**			
·HEMATÍES	6,40	T/L.	5,00 - 8,50 T/L
·HEMOGLOBINA	15,6	g/dL.	12,0 - 18,0 g/dL.
·HEMATOCRITO	44,5	%.	37,0 - 55,0 %.
·VOLUMEN CORPUSCULAR MEDIO	69,5	fL.	60,0 - 77,0 fL.
·HEMOGLOBINA CORP. MEDIA	24,4	pg.	18,5 - 30,0 pg.
·CONC. HEMOGLOBINA CORP. MEDIA	35,1	g/dL.	30,0 - 37,5 g/dL.
·A.DISTR.HEMATIES (RDW)	12,4	%.	< 16,0 %.
**Serie Plaquetar**			
·PLAQUETAS	182	K/uL.	175 - 500 K/uL
·VOLUMEN PLAQUETAR MEDIO	7,4	fL.	5,8 - 9,2 fL.
**Fórmula Leucocitaria**			
·NEUTRÓFILOS	5,7	K/uL.	3,0 - 12,0 K/uL.
·CAYADOS	0,0	K/uL.	< 0,3 K/uL.
·LINFOCITOS	1,9	K/uL.	0,5 - 4,9 K/uL.
·MONOCITOS	0,3	K/uL.	< 2,0 K/uL.
·EOSINÓFILOS	0,1	K/uL.	< 1,5 K/uL.
·BASÓFILOS	0,0	K/uL.	
· % NEUTRÓFILOS	71,4	%.	55,0 - 77,0 %.
· % CAYADOS	0,0	%	< 6,0 %
· % LINFOCITOS	24,0	%.	12,0 - 35,0 %.
· % MONOCITOS	3,9	%.	< 10,0 %.
· % EOSINÓFILOS	0,7	%.	< 10,0 %.
· % BASÓFILOS	0,0	%	< 1,0 %.
FROTIS SANGUÍNEO			

** NO SE OBSERVAN AGREGADOS PLAQUETARIOS.
LA SERIE ROJA Y LA SERIE BLANCA NO PRESENTAN ALTERACIONES MORFOLÓGICAS RESEÑABLES.*

Datos personales

Centro veterinario

Dr. / Dra.

Nº Muestra	Fecha recepción	Fecha impresión	Especie
	**30/08/2024**	**02/09/2024**	**Perro**

PRUEBA	RESULTADO		VAL. DE REFERENCIA
**BIOQUIMICA**			
UREA<br>Espectrofotometría	26	mg/dL.	18 - 60 mg/dL.
CREATININA<br>Espectrofotometría. Límite de referencia actualizado según IRIS 2023	1,19	mg/dL.	0.50 - 1.40 mg/dL.
TRANSAMINASA ALT<br>Espectrofotometría	48	U/L.	10 - 75 U/L.
PROTEÍNAS TOTALES<br>Espectrofotometría	7,0	g/dL.	5,2 - 7,6 g/dL.
**PROTEINOGRAMA ELECTROFORETICO**			
COCIENTE ALBÚMINA-GLOBULINAS	1,41		0,80 - 1,70
ALBÚMINA	4,1	g/dL.	2,3 - 4,5 g/dL.
ALFA 1 GLOBULINAS	0,3	g/dL.	0,2 - 0,5 g/dL.
ALFA 2 GLOBULINAS	0,9	g/dL.	0,3 - 1,1 g/dL.
BETA GLOBULINAS	1,0	g/dL.	0,9 - 2,7 g/dL.
GAMMA GLOBULINAS	0,8	g/dL.	0,5 - 1,2 g/dL.
· % ALBÚMINA	58,5	%.	40,0 - 65,0 %.
· % ALFA 1 GLOBULINAS	4,4	%.	1,0 - 6,0 %.
· % ALFA 2 GLOBULINAS	12,2	%.	5,0 - 15,0 %.
· % BETA GLOBULINAS	13,7	%.	8,0 - 22,0 %.
· % GAMMA GLOBULINAS	11,2	%.	6,0 - 12,0 %.

INMUNOLOGIA

Acs.Leishmania infantum, IgG (A) — NEGATIVO

IFI

** - Negativo ó <1/50: Animal sano. Control anual si no aparecen síntomas compatibles.*

- <1/50: negativo
- 1/50: expuesto (infectado con o sin síntomas)
- 1/100: positivo título bajo
- 1/200: potitivo título moderado
- 1/400 o > 1/400: positivo título alto

Acs. Ehrlichia canis, IgG (A) — NEGATIVO

Method: IFAT

** <1/50: negativo (en animales recién infectados el título de anticuerpos puede ser negativo hasta un mes post-infección)*
1/50: dudoso (puede tratarse del inicio de una seroconversión o de una exposición anterior).
1/100: infección activa (o exposición anterior, no diferencia entre ambas).

ELECTROFORESIS CAPILAR DE PROTEÍNAS SÉRICAS

Date:**02/09/2024**

ID:**0332628**

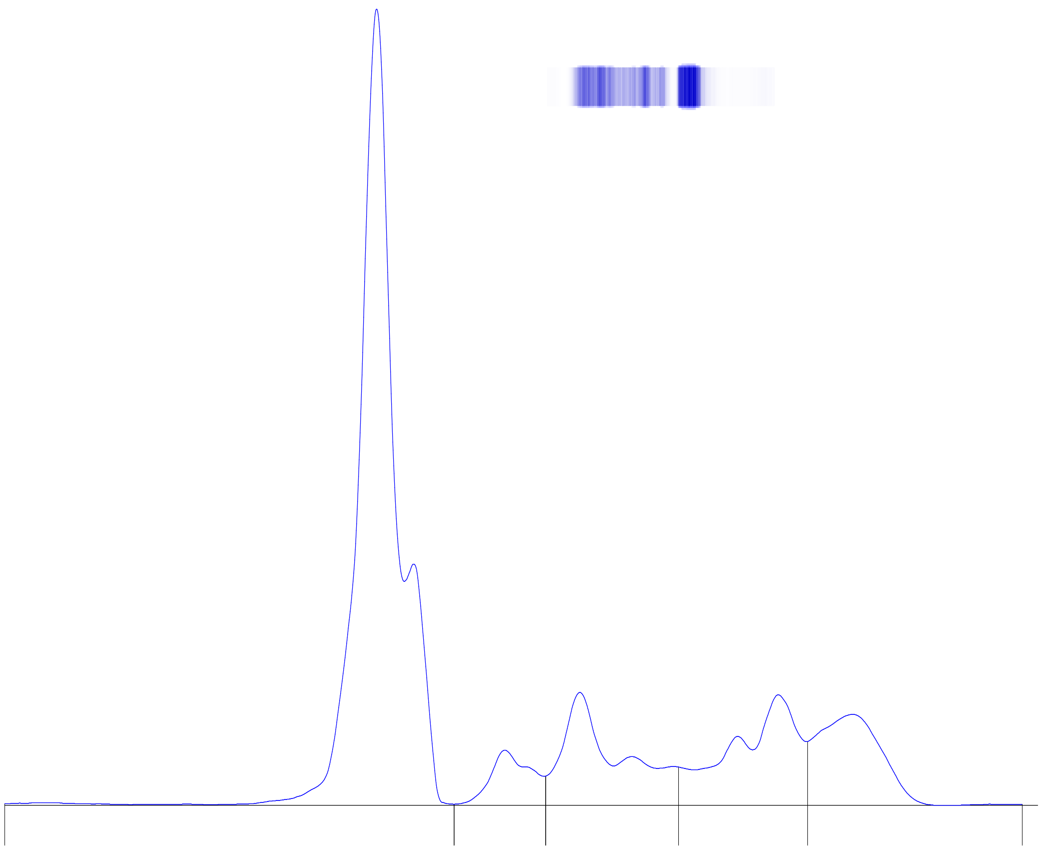

A/G Ratio:**1,41**

Fractions	%
**Albumina**	**58,5**
**Alfa 1**	**4,4**
**Alfa 2**	**12,2**
**Beta**	**13,7**
**Gamma**	**11,2**
